# Tunable Optical Metamaterial Enables Steganography, Rewriting, and Multilevel Information Storage

**DOI:** 10.1007/s40820-025-01897-9

**Published:** 2025-09-05

**Authors:** Jianchen Zheng, Yuzhao Zhang, Haibo Yu, Jingang Wang, Hongji Guo, Ye Qiu, Xiaoduo Wang, Yu Feng, Lianqing Liu, Wen Jung Li

**Affiliations:** 1 State Key Laboratory of Robotics and Intelligent Systems, Shenyang Institute of Automation, Chinese Academy of Sciences, Shenyang, 110016 People’s Republic of China; 2 University of Chinese Academy of Sciences, Beijing, 100049 People’s Republic of China; 3 School of Future Technology, Shanghai University, Shanghai, 200444 People’s Republic of China; 4 Research Center of Micro/Nano-Manipulation, Shanghai University, Shanghai, 200444 People’s Republic of China; 5 Department of Mechanical Engineering, City University of Hong Kong, Hong Kong, 999077 People’s Republic of China

**Keywords:** Micro/nano-device, Optical encryption, Metamaterials, Photoluminescence, 4D printing

## Abstract

In the realm of secure information storage, optical encryption has emerged as a vital technique, particularly with the miniaturization of encryption devices. However, many existing systems lack the necessary reconfigurability and dynamic functionality. This study presents a novel approach through the development of dynamic optical-to-chemical energy conversion metamaterials, which enable enhanced steganography and multilevel information storage. We introduce a micro-dynamic multiple encryption device that leverages programmable optical properties in coumarin-based metamaterials, achieved through a direct laser writing grayscale gradient strategy. This methodology allows for the dynamic regulation of photoluminescent characteristics and cross-linking networks, facilitating innovative steganographic techniques under varying light conditions. The integration of a multi-optical field control system enables real-time adjustments to the material’s properties, enhancing the device’s reconfigurability and storage capabilities. Our findings underscore the potential of these metamaterials in advancing the field of microscale optical encryption, paving the way for future applications in dynamic storage and information security.

**Supplementary Information:**

The online version contains supplementary material available at 10.1007/s40820-025-01897-9.

## Highlights

- Proposed a dynamic grayscale gradient modulation system enabling multi-information analysis and encryption under multi-optical fields, establishing a new paradigm for multi-dimensional encryption of collaborative multispectral information.
- Developed coumarin-based photo-responsive in situ reconstruction technology and constructed a multi-optical field coupled control system to achieve dynamic configuration of multi-information carriers.
- Designed and fabricated a micro-dynamic multiple encryption device with integrated functions for information writing, erasing and rewriting, realizing stable information storage and dynamic destruction through micro/nano-optical keys.

**Supplementary Information:**

The online version contains supplementary material available at 10.1007/s40820-025-01897-9.

## Graphical abstract

**Figure:**
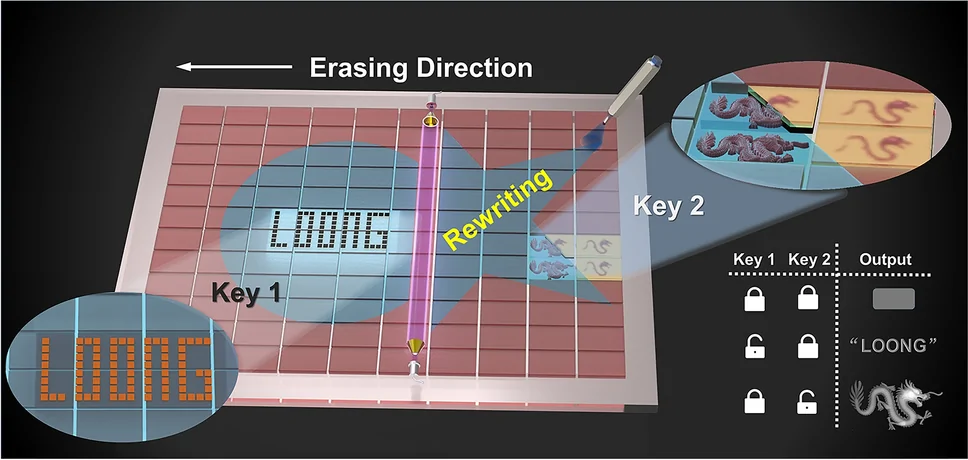

## Introduction

Secure information storage is of great importance not only in the contexts of personal signature authorization, privacy, and information leakage prevention, but it is also crucial on a national level, in terms of currency anti-counterfeiting, military strategies, and international relations [1–3]. Optical information technology, which is also known as optical encryption, is readable by the human eye without sophisticated equipment [4, 5]. However, owing to the tunable degrees of freedom associated with light [6–8], it is possible to change the physical properties of such information in various dimensions to realize encryption [9, 10]. These two unique benefits render optical encryption among the most promising methods for information security storage [11].

Highlights

Over the past few decades, researchers have focused on the development of advanced materials [12–14], the construction of micro- and nano-functional surface structures [15–18], and the modulation of external field systems to realize the encryption storage of optical information [19, 20]. Photoluminescent (PL) materials are commonly used for optical encryption because they can produce varying luminescent colors depending on the light conditions [21, 22]. For example, by combining two organobrominated cuprous hybrids with divergent coordination structures, inkjet printing of materials exhibiting bright cyan and orange emission under different excitations has been achieved [23]. In addition to material innovations, micro- and nano-functional surfaces, such as photonic crystals, surface plasmas [9, 24], and metasurfaces [4, 25], exhibit excellent light manipulation capabilities. They can improve the color gamut, saturation, brightness, thereby serving as candidates for encryption [26]. In addition, changing the lattice constants of three-dimensional (3D) photonic crystals by thermal contraction has achieved 3D structures that range from colorless to vividly colored [27, 28]. Furthermore, embedding magnetic particles in Janus microdroplets has been demonstrated to allow the switching control of the open and closed states of structural colors [29]. Moreover, the optical characteristics of devices have been regulated in terms of the amplitude [30], phase [5], wavelength [31], and orbital angular momentum [32], by the introduction of external field control [33, 34]. With the advent of the information age, traditional encryption devices have failed to satisfy the increasing demands on optical information devices. For example, smaller and finer optical storage devices are required [35, 36], and to ensure information security, multiple encryption strategies must be used in parallel to prevent the finite degree-of-freedom optical passwords from being cracked [22, 37, 38].

Metamaterials/intelligent materials capable of responding to external field excitations in the time dimension can supply new time-dimensional codes for novel optical encryption devices, further reducing the risk of information leakage [17, 39–41]. On the one hand, multiple encryption strategies can be used to split the encrypted information and organically integrate it during reading to prevent forgery and violent decryption. For example, rewriting of information, double encryption [12, 42], and different information outputs under photothermal stimulation [43] were realized using metamaterials. Recently, the researchers fabricated a rewritable three-mode display realized by stimulating interactive fluorescence, phosphorescence, and electroluminescence [44]. In addition, pyrene-based metamaterials can produce information that is automatically erased after a set period of time [45]. On the other hand, researchers continue to break through the minimum processing resolution of the device by continuously improving its processing precision. For instance, the strategy of combining metamaterials with programmed treatment-induced anisotropy via direct laser writing by two-photon polymerization (DLW-TPP) enabled the creation of thermally responsive, reversible graphical displays and concealment at the micrometer scale [46]. While these methods have demonstrated high-security encryption, we propose addressing the existing research gap in miniature encrypted display devices by integrating enhanced reconfiguration precision, faster rewriting speeds, and advanced programmable strategies.

**Fig. 1:**
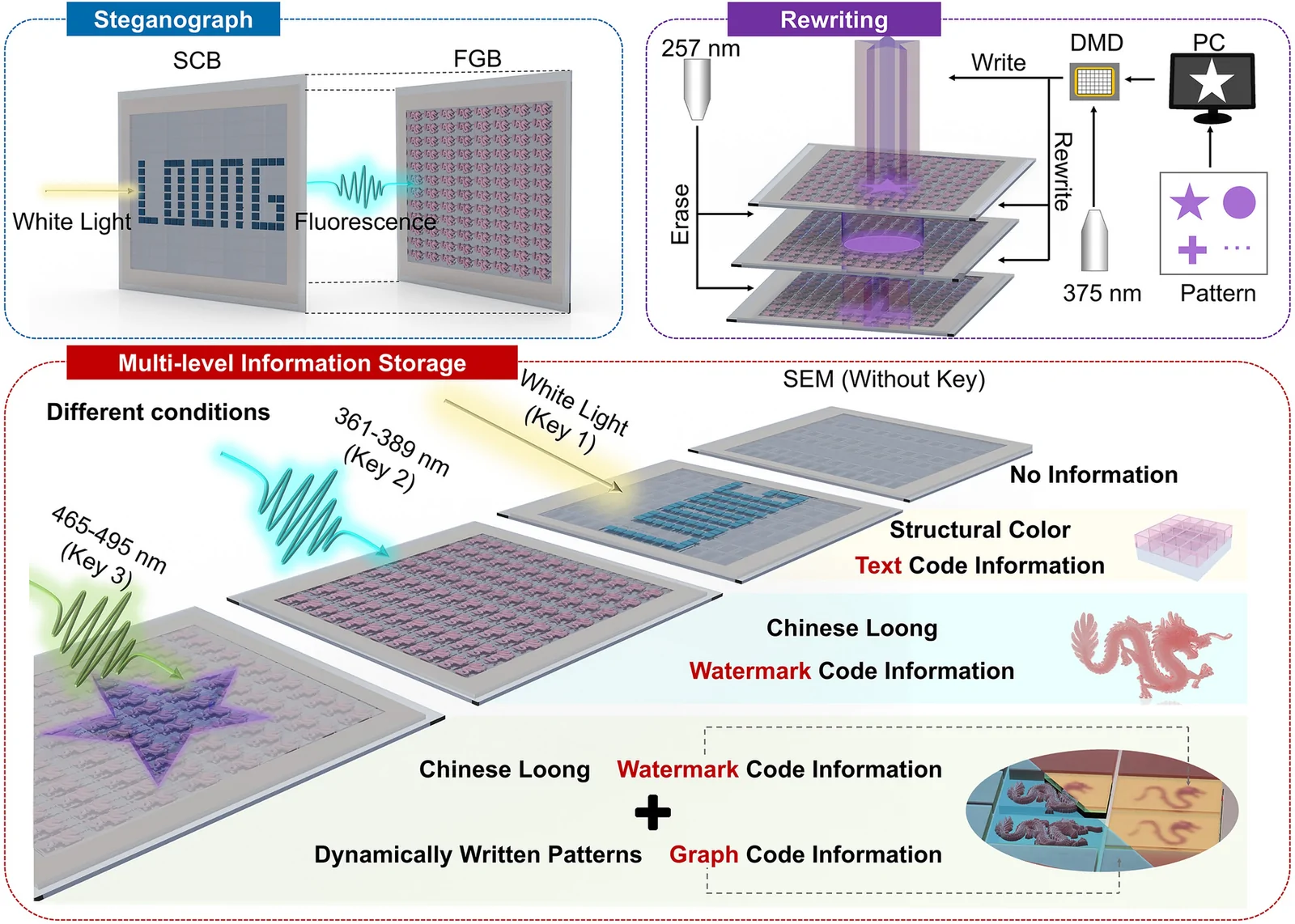
Schematic representation of the micro-dynamic multiple encryptions device (μ-DMED). Two devices based on different photonic microstructures: the fluorescent gray gradient block (FGB) and the structural color block (SCB) are designed for steganography. Light control system implements metamaterials in situ reconfiguration and rewriting of the μ-DMED. Multilevel information, including text, watermark, and graph, is stored under different conditions

In contrast to previous works reporting miniature encrypted devices (Table S1), our work investigates both the dynamically programmable optical information and the individualized reconfigurable design of a device. More specifically, a novel strategy for constructing micro-optical encryption devices using metamaterials that are reconfigurable and dynamically energy conversion programmable is proposed. This approach enables the design and fabrication of a micro-dynamic multiple encryption device (μ-DMED). A combination of direct laser writing (DLW) and grayscale gradient processing strategy is employed to create coumarin functional metamaterials with tunable PL and mechanical characteristics. Subsequently, based on different photonic microstructures, two encrypted devices are designed and fabricated to demonstrate steganography of text and of the “Chinese Loong” pattern under different light fields. To further enhance the dynamic reconfiguration and multilevel storage functions of the device, a synchronized microscopic observation and multi-light field stimulation system is built based on an optical-to-chemical energy conversion mechanism. Under the precise control of the system, the μ-DMED demonstrates the dynamic writing, erasing, and rewriting of different patterns, and the synchronized multilevel information storage of “Chinese Loong” watermarks and pattern information, as illustrated in Fig. 1. The designed μ-DMED shows promising prospects for next-generation advanced cryptographic devices with its flexible dynamic reconfigurability and multilevel information storage.

## Experimental Section

### Preparation of the Precursor Solution

AAc (0.4 mL, 99%, Aikeshiji), ATC (0.475 g, self-configuration [47]), and polyvinylpyrrolidone (0.75 g, PVP, Mw 1,300,000, Aladdin) were combined in ethyl lactate (5 mL, EL, 98%, 9DingChemistry). An aliquot (2.5 mL) of this solution was then mixed with DPEPA (0.4 mL), TEA (0.5 mL), and EMK/DMF (100 μL, 20 wt%) and stirred overnight. The above process was carried out in a yellow light environment.

### DLW-Based Microstructural Processing

All microstructures were processed using a commercial two-photon polymerization system (Photonic Professional GT2, Nanoscribe GmbH, Germany). A 63 × oil immersion objective (NA = 1.4, Zeiss, Germany) was used throughout. The configured precursor solution was placed on the top layer of a cover glass (*d* = 30 mm, Thermo Fisher Scientific). The bottom layer consisted of the oil (Immersol 518F, Zeiss, Germany) required to submerge the immersion objective. The 3D structures were designed in SolidWorks and then converted to STL files and adapted for fabrication with Nanowrite software on a femtosecond laser direct writing processing system. 3D micro- and nanofabrication of the samples was performed using the TPP technique. The preset microstructure morphology was constructed by layer-by-layer exposure, and we set the angle offset to 90° and the hatching distance to 0.1 μm. The system was then processed using a 780 nm femtosecond laser in a stable temperature (22 °C) and humidity (40%) environment. All experiments were performed using the Galvo scan mode for microstructural processing. The processed structures were submerged in isopropyl alcohol (IPA, 99.9%, Aladdin) for at least 1 h, with one solution change during this time to remove any excess precursor solution.

### Characterization

SEM images were obtained using a Quattro S instrument (Thermo Fisher Scientific, USA). The fluorescence properties of the microstructures were investigated using an FS920P fluorescence spectrometer (Edinburgh Instruments, UK) and a Raman-LS6 spectrometer (HORIBA Scientific, Japan). The viscoelastic curves were obtained using a Bruker Dimension Icon AFM (Bruker, Billerica, MA, USA). The optical images were captured using an Eclipse Ti microscope (Nikon). The fluorescence images were captured using an Eclipse Ti microscope (Nikon, Japan) and a confocal microscope (A1R MP, Nikon, Japan). The surface topography was measured by a 3D surface profiler based on scanning white light interferometry (Contour FTK-A, Bruker, USA). The microstructure was characterized by micro-Fourier-transform infrared (Micro-FTIR) spectroscopy (Nicolet iN10, Thermo Scientific, USA). The spectra were obtained in the range of 4000–400 cm^−1^ with 30 scans and a resolution of 4 cm^−1^ at 25 °C.

### System Construction

The synchronized observation and external excitation system consisted of a 375 nm UV laser (MW-UV-375, Changchun Femtosecond Technology Co., Ltd., China), a 257 nm UV laser (MPL-N-257, Jilin RAMCO Optical Equipment, China), a DMD (D4100-7 XGA Kit, Texas Instruments, Inc., USA), an optical lens set, a reflector, a mobile platform, and a CCD (Osmosis Micro, China). The optical lens set was composed of plano-convex and plano-concave lenses, which were coated with a 350–700 nm transmittance enhancement film to minimize attenuation of the UV light passing through the lens. The focal lengths of the plano-concave and plano-convex lenses were 50.2 and − 30.0 mm, respectively. By adjusting the distance between the two lenses to make the focal points coincide, the beam can be narrowed without affecting the parallelism of the incident light. The DMD was composed of 1024 × 768 micromirrors, each measuring 13.6 μm × 13.6 μm and being individually adjustable to obtain a patterned micromirror structure and reflect the incident light back in a specified pattern by adjusting some micromirrors. A 10 × objective lens with a numerical aperture of 0.25 and a 325–500 nm transmittance enhancement coating was used for focusing. A dichroic mirror (DM10-490SP, LBTEK, China) and a reflector were used to regulate the two laser beams in the same vertical plane.

### Data Analysis

Spectral data filtering was performed using the PyWavelets toolkit in Python. Wavelet analysis methods were used to denoise and smooth the signals, and the raw signals were processed using the db4 wavelet basis, soft thresholding, and the 6-layer decomposition method. The data were processed and replotted using the subtracted baseline in the Analysis Baseline of OriginLab 2024 (USA).

### Numerical Simulation

We performed the spectral analysis of the SCB using the finite-difference time-domain (FDTD, Lumerical) method, employing a plane wave source as the light source. The wavelength range of the source was set from 300 to 800 nm. Periodic boundary conditions were applied in the *X* and *Y* directions, while an ideal perfect matching layer (PML) was used as the boundary condition in the *Z* direction. The refractive index of the color block material was set to 1.56, and the refractive index of air was taken as 1.0.

## Results and Discussion

### Properties Tuning of the Metamaterials

**Fig. 2:**
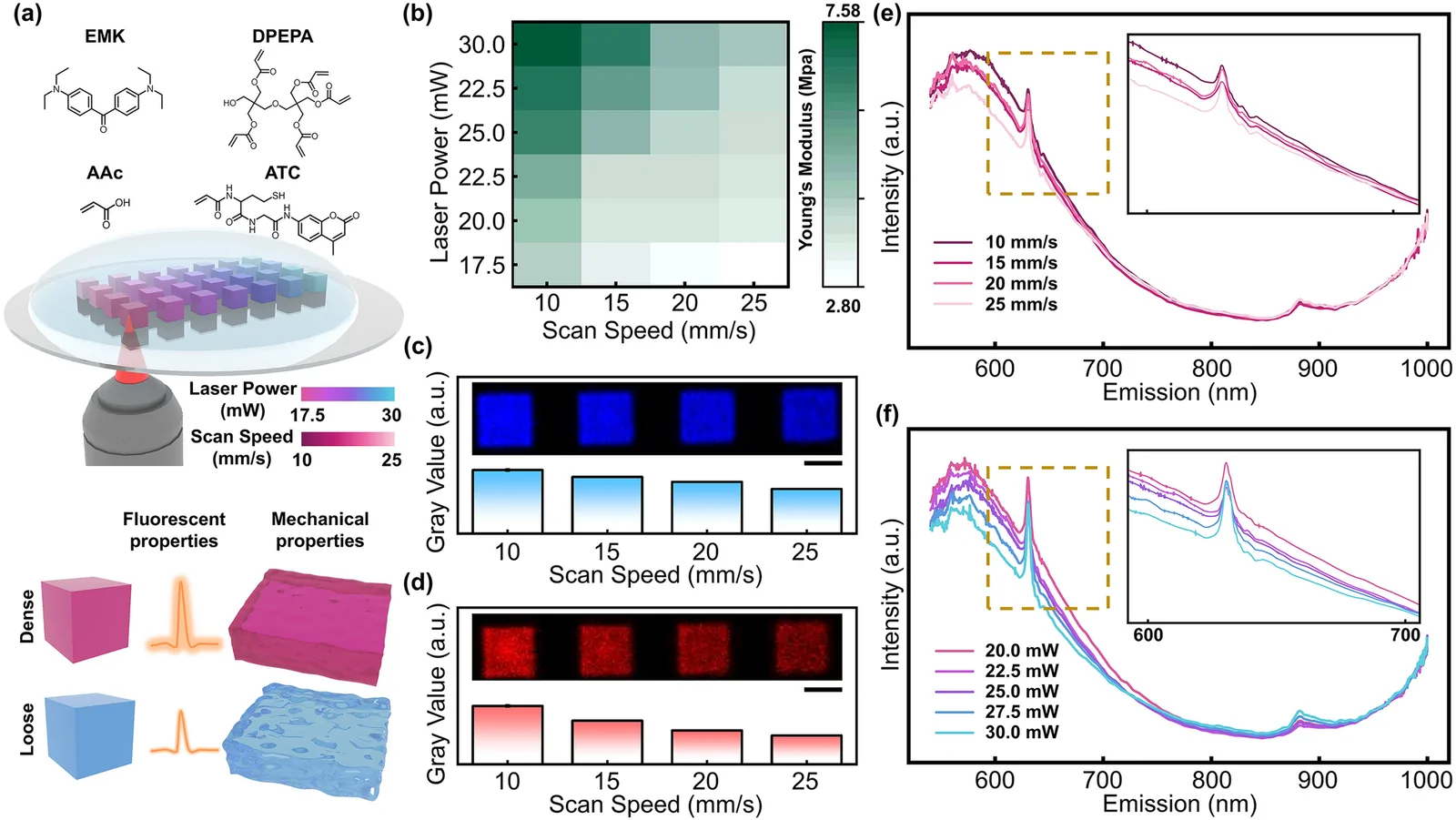
Strategy for programmable processing and property modulation of the microstructure. **a** Schematic representation of the DLW-based approach for microscale processing and property modification. **b** Relationship between the laser processing parameters and the Young’s modulus. **c**, **d** Effect of the scan speed on the fluorescence intensity. LP = 30 mW. **c** ${\lambda }_{ex}$ = 405 nm, ${\lambda }_{em}$ = 425–475 nm. **d** ${\lambda }_{ex}$ = 640 nm, ${\lambda }_{em}$ = 663–738 nm. Shifts of the emission spectra under 532 nm laser excitation with variation in **e** scan speed, and **f** laser power. Scale bars: 10 μm

Initially, the precursor solution required for DLW processing was configured. This solution consisted of 4,4′-bis(diethylamino)benzophenone (EMK) as a photoinitiator, dipentaerythritol pentaacrylate (DPEPA) as a cross-linking agent, and acrylic acid (AAc) and acrylamide–thiol–coumarin (ATC) as two functional monomers. Notably, incorporation of the ATC monomer resulted in light-responsiveness and PL properties in the processed microstructures. During DLW processing, the laser-scanned area was rapidly shaped, and a 3D microstructure was obtained, as shown in Fig. 2a.

Subsequently, the effects of different processing parameters on the cross-link density of the internal microstructure network were evaluated. It was found that as the laser power (LP) was increased and scan speed (SS) was decreased, the network became denser. In contrast, decreasing the LP and increasing the SS loosened the network (Fig. S1). Importantly, variation in the cross-link density can be used to modulate the properties of microstructures. Thus, the effects of different processing parameters on the mechanical and PL properties of the microstructures were examined [46]. For this purpose, 30 μm × 30 μm × 5 μm (*L* × *W* × *H*) arrays of microblocks were prepared using SSs ranging from 10 to 25 mm/s and LPs ranging from 17.5 to 30 mW. The force profiles of the microstructures were tested in air using atomic force microscopy (AFM) to obtain the Young’s moduli under different processing parameters (Fig. S2), wherein a higher Young’s modulus reflects a higher rigidity (Fig. 2b). It was found that the Young’s modulus increased with an increase in the LP at a constant SS. In contrast, when the LP was fixed, the Young’s modulus of the microstructure decreased as the SS was increased. It was therefore apparent that upon reducing the SS or increasing the LP, the network inside the microstructure becomes denser, and the rigidity of the microstructure increases, and vice versa.

Owing to the large number of coumarin functional groups present in the material, the microstructures exhibited PL properties, and so the relationships between the fluorescence function and various processing parameters were explored. Figure 2c and d shows the fluorescence images of the microstructures in different wavelength ranges and under different excitation light sources for a LP of 30 mW. The gray values of the fluorescence region, which represent the fluorescence intensity in the current excitation state, were further measured, and the results showed that the fluorescence intensity was inversely proportional to the SS and directly proportional to the LP (Fig. S3). Subsequently, the emission spectra of the microstructures with different processing parameters under an excitation of 532 nm were further examined (Fig. 2e, f), and it was found that the different processing parameters did not affect the shape of the microstructure emission spectra, but they had significant effects on the fluorescence intensity.

### Information Steganography Based on the Gray Gradient Strategy

**Fig. 3:**
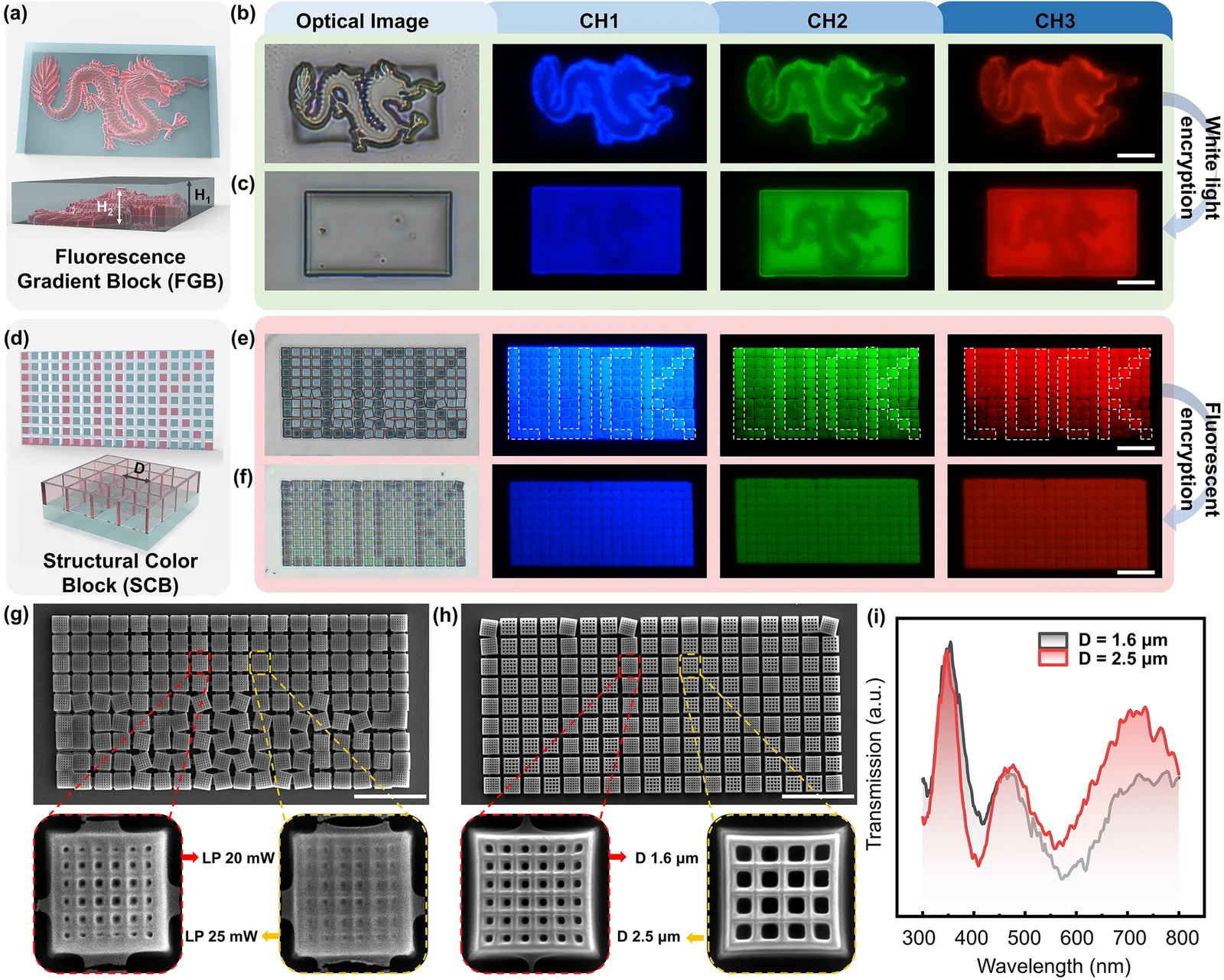
Steganography strategies based on fluorescence tuning. **a** Design of fluorescence gradient blocks (FGBs) based on white light encryption. **b** Optical and fluorescence images of the non-encrypted FGBs when *H*_1_ < *H*_2_. **c** Optical and fluorescence images of the white light encryption FGBs when *H*_1_ > *H*_2_. **d** Design of structural color microblocks (SCBs) based on fluorescence encryption. **e** Non-encrypted devices of the same SCB design under variable processing parameters. **f** SCB fluorescent encrypted devices with variable model parameters for the same processing parameters. **g**, **h** SEM images corresponding to parts (**e**, **f**), respectively. Scale bars: 20 μm. **i** Spectral FDTD simulations corresponding to different structural parameters of the SCBs. CH1: ${\lambda }_{ex}$ = 361–389 nm, ${\lambda }_{em}$ = 430–490 nm. CH2: ${\lambda }_{ex}$ = 465–495 nm, ${\lambda }_{em}$ = 512–558 nm. CH3: ${\lambda }_{ex}$ = 510–560 nm, ${\lambda }_{em}$ = 580 nm

Considering the tunable optical properties of the prepared microstructures, it should be possible to decode and encrypt complex graphical and textual information to produce highly secure systems. As shown in Fig. 3a, a fluorescent gray gradient block (FGB) was designed based on the programmable PL properties to obtain steganography devices under white light conditions. The “Chinese Loong” pattern was used as the graphical information for encryption, and the outer encrypted block structure (70 μm lateral dimension) was embedded on the surface of the pattern. The processing parameters of the outer encryption block were set to ensure a sharp contrast between the pattern and the encryption block, with the aim of guaranteeing display clarity while still providing structural stability (Fig. S4). The parameters of *H*1 and *H*2 can be 3D sliced and scanned layer by layer in Nanowrite software according to the set height and printing parameters, respectively (Fig. S5a-h). Here, the processing parameters of the encryption layer and the pattern layer were maintained at LP = 30 mW, SS = 10 mm s^−1^ and LP = 17.5 mW, SS = 20 mm s^−1^, respectively. Notably, the different embedding heights of parameters *H*1 and *H*2 directly affected the degree of encryption. More specifically, when *H*1 was set to 1 μm and *H*2 was set to 5 μm, the FGB exhibited an unencrypted state. Optical and fluorescence images of the different channels (CHs) are shown in Fig. 3b, wherein it is apparent that the Chinese Loong pattern can be clearly observed under both white light and fluorescence conditions for the different CHs. In contrast, with *H*1 = 6 μm and *H*2 = 5 μm (Fig. 3c), the FGB exhibited encryption under white light illumination. Furthermore, the readout of the Chinese Loong pattern under different fluorescence conditions showed obvious differences, among which the CH2 condition led to the clearest display results. Moreover, the 3D surface profiler with scanning electron microscopy (SEM) results show both readable (Fig. S6a) and unreadable (Fig. S6b) encryption modes. Only the flat surface of the encrypted rectangular block was observed in the encrypted mode, whereas the internal Chinese Loong pattern was completely hidden.

Based on our previous exploration of structural color blocks (see Fig. S7 for the structural color block model) [47], an optically steganography device consisting of 18 × 9 structural color block (SCB) units was fabricated (detailed process shown in Movie S1), wherein the dimensions of a single SCB were 3 μm × 3 μm × 3 μm (*L* × *W* × *H*). In this case, the textual information to be encrypted and the non-encrypted background information are composed of different SCBs, being programmed either to fix different processing parameters or change different grid distances (*D*), as presented in Fig. 3d. The dimensions of the encrypted display device were approximately 110 μm × 55 μm × 6 μm (*L* × *W* × *H*). When the information text “LUCK” was presented by the background and the text with identical *D* values but different processing parameters (Fig. 3e), the text was legible under various light field conditions. Upon altering the *D* of the background and text SCBs under constant processing parameters, the text was only legible under white light conditions, but remained unreadable and encrypted under fluorescence conditions (Fig. 3f). Further SEM observations (Fig. 3g, h) demonstrated that the text information in both situations remained legible. Besides, we further simulate the spectra of SCBs with different structures by FDTD simulation. The results showed that the simulation spectral results (Fig. 3i) had a similar trend with the corresponding transmission spectra (Fig. S7d), and the spectral color differences between the two-color blocks could be clearly separated. The experimental results also confirmed the detailed structural features of the two encrypted device backgrounds containing text SCBs. Importantly, using the proposed fabrication strategy, the readable display and encryptable hiding functions of graphics and text under different conditions were statically demonstrated. Notably, the two cryptographic devices measured no more than ~ 100 μm, offering a new paradigm for advanced optical display and encryption at the microscale.

### A Multi-light-Field Coupled In Situ Control System Based on Light Energy Conversion

As described above, the presence of abundant coumarin groups in the cross-linked network led to the generation of a light-responsive microstructure. In the coumarin structure, the double bond and the conjugated electrons of the benzene ring are delocalized, and under 375 nm irradiation, the double bonds of two molecules can undergo a [2 + 2] cycloaddition reaction to form a coumarin dimer, thereby increasing the cross-link density of the network. While under 257 nm irradiation, photocleavage of the coumarin dimer occurs, returning to the double bond state, and reducing the cross-link density. This light response is reversible and represents the conversion of light energy into chemical energy to dynamically adjust the network cross-linking inside the microstructures.

**Fig. 4:**
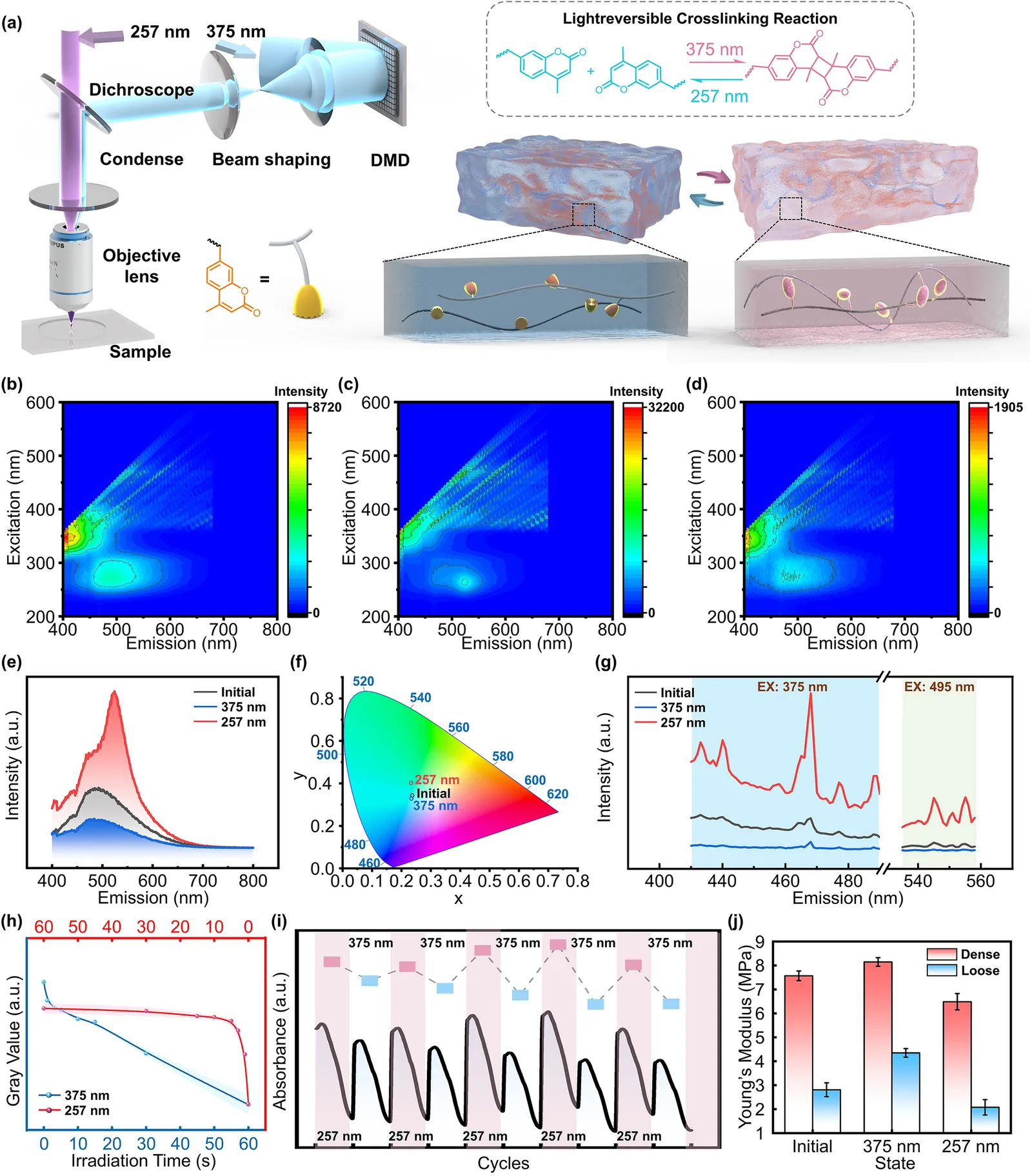
Dynamic light energy conversion in metamaterials. **a** Optical regulatory systems and dynamic regulatory mechanisms. Excitation–emission–matrix (EEM) spectra before and after light energy conversion; **b** initial state **c** after 257 nm laser irradiation, and **d** after 375 nm laser irradiation. **e** Emission spectra of the different states under 272 nm light excitation. **f** Coordinate positions of CIE 1931 corresponding to the three states presented in part **e**. **g** Emission spectra in specific ranges at different excitation wavelengths for the three states. **h** Changes in the fluorescence intensity with time after light irradiation at different wavelengths. **i** Cycling of its light absorption curve from 300 to 340 nm under repeated experiences of different laser irradiation. Where the square represents the absorbance at 320 nm. **j** Changes in the mechanical properties of the loose and dense layers before and after light energy conversion

To achieve dynamic reconfiguration and rapid energy conversion of the materials, a multi-light-field coupled in situ control system (MICS) was constructed to synchronize the observation and excitation of light stimuli, as shown in Fig. 4a. This system combined 375 and 257 nm ultraviolet (UV) lasers, aligning the light paths of the two laser beams in the vertical plane using a dichroic mirror and a reflector. The shaped laser beam was focused onto the sample surface using a focusing lens and a 10 × objective of 0.25 numerical aperture. In the 375 nm UV light path, the beam-shaping lens set was integrated with a digital micromirror device (DMD), enabling the maskless pattern to be projected onto the sample surface. The imaging objective and CCD camera were inverted under the sample, enabling real-time acquisition of the sample image and dynamic feedback of the spot irradiation area (Fig. S7). This system allowed dynamic light energy conversion and real-time observation of the sample surface. In this study, 375 and 257 nm lasers were used with irradiation powers of 20 and 30 mW, respectively.

### Tunable Dynamic PL Characteristics Based on MICS’ Energy Conversion

The MICS is able to dynamically regulate the PL properties of microstructures. Thus, experiments were carried out to analyze the changes in fluorescence spectra after light energy conversion. A series of 30 μm × 30 μm × 5 μm (*L* × *W* × *H*) square arrays with a pitch of 10 μm were processed with an LP of 22.5 mW and an SS of 10 mm s^−1^. The fluorescence spectra and intensities of the microcube arrays in the different states were analyzed, wherein Fig. 4b–d shows the excitation–emission–matrix (EEM) spectra of the microstructure before irradiation, after 257 nm irradiation, and after 375 nm irradiation, respectively. The results showed a significant increase in the fluorescence intensity after 257 nm irradiation and a decrease in the fluorescence intensity after irradiation at 375 nm.

In order to further demonstrate the mapping relationship between coumarin chemical cross-linking and photophysics properties, micro-FTIR was used to characterize the transmittance of a square structure with a side length of 30 μm. Comparison of the infrared spectra of the microstructures irradiated at 375 and 257 nm reveals that the C=C (~ 1600 cm^−1^) telescopic vibrational peak of the coumarin parent nucleus is weakened or disappeared under 375 nm irradiation, and a distinct characteristic peak, which is cyclobutane-derived, appears at ~ 900–1000 cm^−1^. This n-butane originated from the C=C of coumarin underwent [2 + 2] cycloaddition (Fig. S9).

Furthermore, the emission spectra of the three states under 272 nm light excitation were analyzed, and it was apparent that the intensity for the sample subjected to 257 nm irradiation was significantly larger than those observed for the other two states (i.e., initial and after 375 nm irradiation), as shown in Fig. 4e. In addition, Fig. 4f shows the color positions of CIE1931 corresponding to the three spectra presented in Fig. 4e. The obtained results confirm that the colors in the initial state and after 375 nm irradiation are similar, and that the spectral intensity is not only enhanced after irradiation beyond 257 nm, but that it is also shifted toward the green region. Moreover, the spectrum of the initial state is closer to that after 375 nm irradiation, which may be due to the fact that a 780 nm laser was used during DLW processing; at this wavelength, both cross-linking and simultaneous photodimerization of the coumarin groups are promoted.

To analyze the fluorescence change state of the metamaterials under microscopy, the fluorescence spectra of the microstructures under two different excitation lights in specific emission regions are extracted, as shown in Fig. 4g. The blue region with an excitation wavelength of 375 nm refers to CH1 in Fig. 3, whereas the green region, with an excitation wavelength of 495 nm, refers to CH2. The results show that the change in intensity after irradiation with CH1 was more obvious than that with CH2. Subsequently, the changes in the fluorescence intensity under repeated CH1 irradiation at 375 and 257 nm were analyzed individually; the gray values of the fluorescence images with continuous changes were extracted, and the change curves of the fluorescence intensity were obtained over 60 s, as shown in Fig. 4h. It was found that the fluorescence intensity of the structure showed a linear decrease after irradiation at 375 nm, whereas after irradiation at 257 nm, the fluorescence intensity rapidly increased and gradually reached a steady state after irradiation for 10 s. However, the fluorescence intensity was slightly depleted relative to the initial state.

Since the switching characteristic of coumarin mainly occurs at the 320 nm peak, we tested and calculated the variation of its light absorption curve from 300 to 340 nm under repeated experiences of laser irradiation. As can be observed in Fig. 4i, the characteristic peak decreased when subjected to 375 nm laser irradiation and increased when subjected to 257 nm laser irradiation. In addition, the results of fluorescence grayscale characterization of the structure (Fig. S10) also show corresponding results: The fluorescence intensity decreases after 375 nm irradiation; in contrast, the fluorescence intensity increases after 257 nm irradiation. After approximately 20 cycles, the device’s PL dynamic switching capability diminishes, resulting in minimal changes to the characteristic peak. These results indicate the potential of this system to personalize encryption devices.

As shown in Fig. 4j, the mechanical properties of the microstructure changed upon the conversion of light energy. More specifically, after irradiation at 375 nm, the sample stiffness was greater than that after irradiation at 257 nm, regardless of whether the layer was dense or loose. This was attributed to the fact that the photodimerization taking place after 375 nm irradiation produces a denser internal network, which results in a larger Young’s modulus for the microstructure. After 257 nm irradiation, the internal network undergoes photocleavage, the network becomes loose again, and the Young’s modulus decreases accordingly.

### Reconfigurable Storage–Erasing–Rewriting of Multilevel Information

**Fig. 5:**
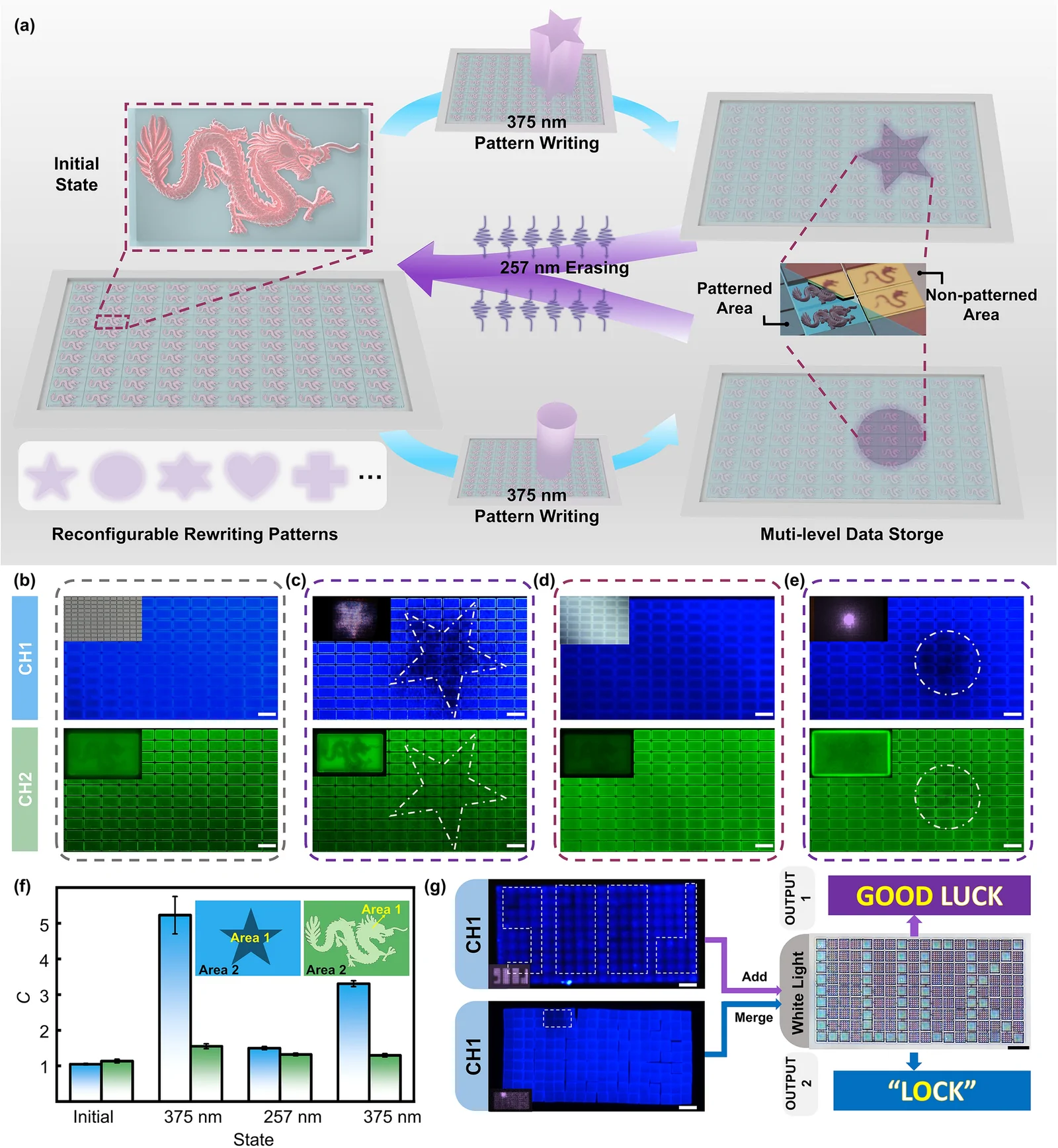
Reconfigurable storage–erasing–rewriting of multilevel information μ-DMED. **a** A schematic diagram of the μ-DMED based on FGBs. **b** Fluorescence diagram of the FGB device in the initial state. **c** Patterned writing and multilevel storage after 375 nm star spot irradiation. **d** Patterned erasure after 257 nm irradiation. **e** Circular patterned rewriting after 375 nm spot irradiation. **b–e** Scale bar: 70 μm. **f** Contrast change in the FGB device with different light irradiation. **g** Demonstration of multiple encryption modes for SCB-based μ-DMED. Scale bar: 20 μm

Based on the above tunable characterization of metamaterials, micro-dynamic multiple encryptions devices (μ-DMEDs) can be designed, enabling different light writing**–**erasing–rewriting, and multilevel information storage (including watermark and reconfigurable writing patterns), as shown in Fig. 5a. To demonstrate such functionality, we designed two different types of μ-DMEDs. Firstly, 12 × 11 FGBs were used to produce a μ-DMED (Movie S2) measuring 950 μm × 540 μm (*L* × *W*; Fig. 5b). The writing of pattern information (Fig. 5c), in situ pattern erasing (Fig. 5d), and pattern rewriting (Fig. 5e) were realized by external optical stimulation using the MICS. In the initial state (Fig. 5b), the Chinese Loong watermark stored in each FGB. The contrast of the FGBs under CH2 conditions was sharper and presented a clearer watermark pattern. Furthermore, when a pentagram spot (outer circle diameter 500 μm) was irradiated through the DMD to the μ-DMED (irradiation time = 60 s), the external light energy was converted to internal chemical energy, thereby leading to a change in performance under different light field conditions.

Interestingly, it was found that laser irradiation does not cause a change in the encryption state of the μ-DMED under white light conditions. As shown in Fig. 5c, the intensity of the fluorescence image in pattern area for CH1 decreased remarkably after 375 nm laser irradiation, enabling the formation of a pentagram-shaped writing. The illustration shows the geometry of the laser spot during irradiation; wherein partial scattering of the laser spot results in non-uniform energy irradiation on the μ-DMED. In the case of CH2, no sharp pentagram shape was produced within the irradiation region. This phenomenon was attributed to the differences in fluorescence intensity after irradiation for the two channels, which is consistent with the results of previous spectral analyses. Notably, the watermark clarity inside the irradiated region for CH2 is slightly enhanced.

Following 257 nm laser irradiation of the μ-DMED surface for 60 s (Fig. 5d), the pattern on the μ-DMED surface at CH1 was almost entirely erased; the inset shows the area irradiated by the laser. The clarity of the watermark within CH2 was slightly reduced, along with the overall fluorescence intensity. In dynamic rewriting stage, it can be seen from Fig. 5e that when the circular spot pattern is irradiated onto the μ-DMED surface once again (the inset shows the shape of the spot), the rewritten circular pattern (~ 200 µm diameter) is obviously visible in CH1. These results demonstrate that the sharpness of the watermark improved in CH2. In addition, laser irradiation did not alter the encryption of the μ-DMED under white light conditions; multilevel information remained unreadable.

To quantitatively describe the differences in the graphical information after laser irradiation, the clarity and average fluorescence gray values were used to quantitatively depict the results of the above experiments. Thus, the clarity (C) is defined as:

1 $$C=\frac{{G}_{2}}{{G}_{1}}$$

where G is the average gray value of the region. As shown in the inset of Fig. 5f, ${G}_{1}$ in CH1 represents the irradiated region and ${G}_{2}$ refers to the unirradiated region. In CH2, ${G}_{1}$ and ${G}_{2}$ represent the bright and dark regions, respectively, within a single FGB. Initially, the clarity changes of the FGB-based μ-DMED write–erase–rewrite process were evaluated, as shown in Fig. 5f. After writing the pattern, the clarity of CH1 was improved by ~ 5.2 times, while the clarity of CH2 was enhanced by 0.37 times. Following erasure of the data, the clarity of CH1 recovered to 1.49, while that of CH2 decreased to 1.32. Rewriting the pattern resulted in another clarity improvement of 3.3 times for CH1 relative to the initial state, although no significant change was observed for CH2.

Subsequently, as shown in Fig. 5g, multiple encryption modes based on μ-DMEDs with SCBs were also demonstrated. More specifically, the pattern was written into the μ-DMED after irradiation with a 375 nm “GOOD” shaped spot for 60 s. At this time, the first password output “GOOD-LUCK” message was obtained by adding the text message in CH1 and the text message in the white light of the μ-DMED. After irradiating the 375 nm rectangular light spot for 60 s, the second type of cryptographic output “LOCK” can be obtained by merging the information in CH1 with the text information of μ-DMED under white light (Fig. S11).

**Fig. 6:**
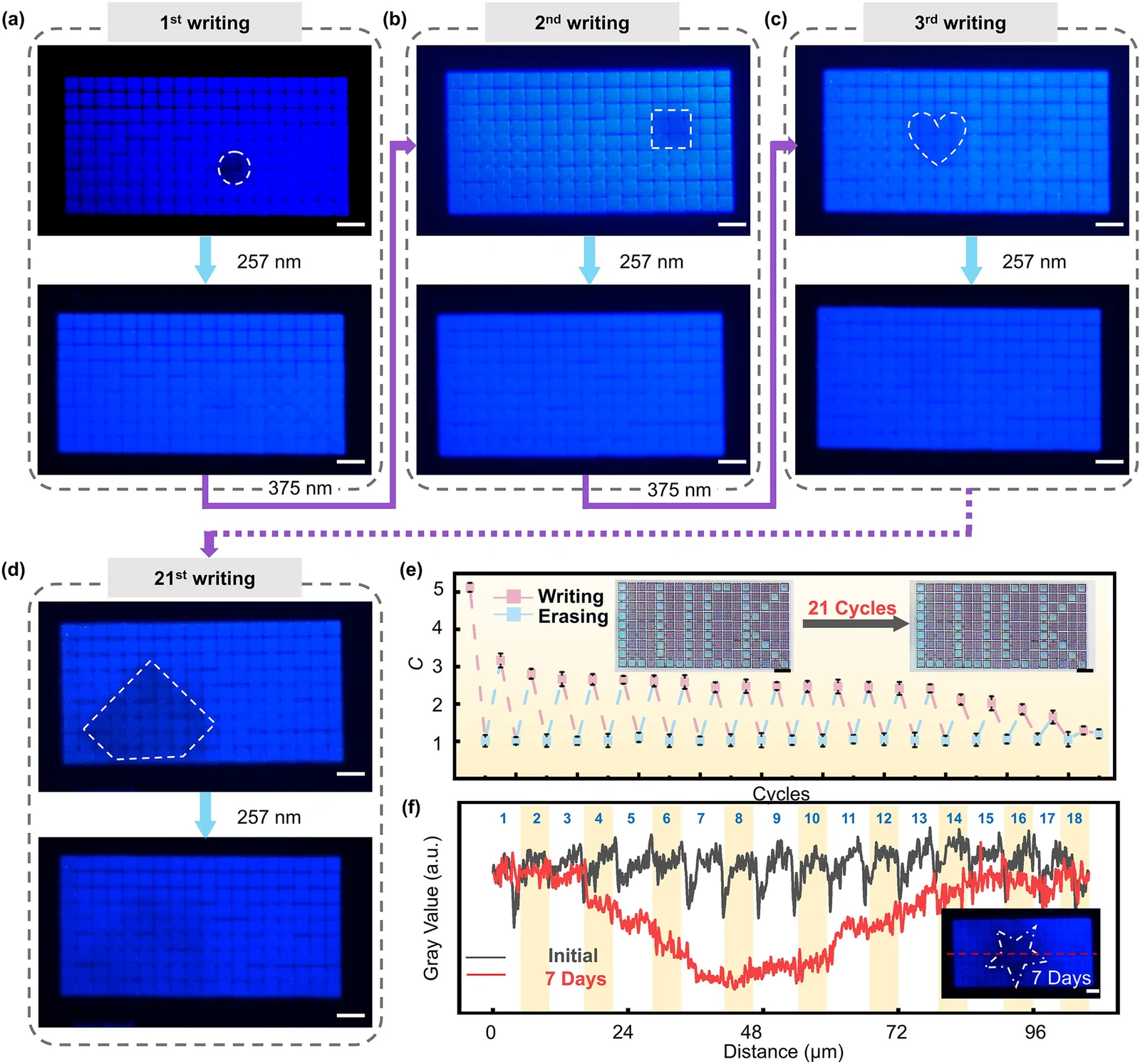
Stability testing of μ-DMED. **a–d** Erase–rewrite repetitive stability performance test of μ-DMED for in-cycle graphics. **a**,** b**,** c**, and **d** correspond to the first, second, third, and 21st erase–rewrite processes, respectively. **e** Contrast and white light image variation of μ-DMED over 21 cyclic erase–rewrite cycles. **f** Grayscale values of the fifth row of SCB-based μ-DMED (red dashed row in the inset) after 7 days of irradiating the pentagram pattern. All the scale bars are 20 μm

In addition, the cyclic repeatable write–erase capability of the device is critical for its long-term stable storage and use. Therefore, we tested the graphic information write–erase capability of μ-DMED within 21 cycles. As shown in Fig. 6a–c, we irradiated the μ-DMED for 60 s with circular-, rectangular-, and cardiac-shaped 375 nm spots, and the patterns were clearly written on the μ-DMED. After undergoing 257 nm laser irradiation, the written information can be erased and prepared for the next rewrite. The device achieves stable erase–rewrite capability for 20 cycles. After the 21st cycle of writing the pentagonal pattern, irradiating the μ-DMED with a 257 nm laser, the pentagonal pattern could not be completely erased (Fig. 6d), and the device’s rewrite capability failed.

As shown in Fig. 6e, we further characterized the change in contrast of μ-DMED over these 21 cycle times. After the initial pattern writing, the contrast reaches 5.17, while the pattern contrast tends to decrease during the 375 nm laser cycle writing. Moreover, the erasing ability of 257 nm gradually decreases. Eventually, after 21 cycles, the μ-DMED’s erasing ability almost fails. Notably, the SCB text message “LUCK” under white light conditions proved to be clearly readable after repeated irradiation (Fig. 6e illustration). Furthermore, Fig. 6f shows that the pattern is clearly written on the μ-DMED after 60 s of 375 nm irradiation by a pentagram spot (outer circle diameter = 60 μm).

The long-term preservation capability of this SCB-based μ-DMED graphical information writing was further analyzed, in which the fluorescence gray value curve of the fifth row of the μ-DMED (red dashed row, Fig. 5h), which had been preserved for 7 days, was compared with the curve of the same row before writing. More specifically, before writing, each SCB unit possessed relatively homogeneous grayscale values. In contrast, after writing of the pentagram pattern, the grayscale values showed a noticeable decreasing trend from approximately the fifth SCB unit, along with an increasing trend at the twelfth SCB unit, coinciding with the experimentally irradiation region.

Not only that, the spectral graphic of the SCB text information stored under white light conditions after 10 days (Fig. S12) shows that there is almost no significant change in the text information compared to the pre-storage period, proving the stable storage and reading ability of the information under white light conditions. Figure S13 also shows the graphical and textual information stored under CH1 and white light conditions, respectively, demonstrating that the μ-DMED exhibits a reliable performance, stably preserving multilevel information for the long term. The above analysis therefore clearly demonstrates that the μ-DMED exhibits erasable and rewritable functions, presenting high-security, programmability, and multilevel information storage stability, thereby offering a novel pathway for the preparation of devices for microscale individualized encryption and anti-counterfeiting technology.

## Conclusions

In conclusion, this work innovatively develops a micro-dynamic multiple encryptions device (μ-DMED) with integrated encryption, rewriting, erasing, and storage properties. Through synthesizing optical-to-chemical energy conversion metamaterials, the relationship between laser writing parameters and microstructural properties (e.g., photoluminescence and mechanical properties) was thoroughly investigated. Furthermore, a grayscale gradient-tunable method was introduced to construct two encryption devices: fluorescent gray gradient block (FGB) and structural color block (SCB), demonstrating remarkable steganography capabilities under various illuminations. Coumarin-based in situ metamaterial reconstruction and energy conversion via a multi-field coupled in situ control system (MICS) with the ability to dynamically regulate microstructural photoluminescence. Additionally, two μ-DMEDs were developed for dynamic information writing/erasing/rewriting with long-term multilevel storage stability, highlighting their potential in dynamic encryption, energy conversion, and microscale storage. The constructed μ-DMED demonstrated outstanding overall performance, including dynamic reconfigurability, high precision (700 nm), rapid rewriting (60 s), stability, and exceptional graphic printing capability, surpassing similar micro-optical encryption devices (as shown in Table S1). These findings pave the way for the future development of next-generation microscale cryptography and smart materials.

## Supplementary Information

Below is the link to the electronic supplementary material.

Supplementary file1 (DOCX 6224 kb)

Supplementary file2 (MP4 2338 kb)

Supplementary file3 (MP4 3097 kb)
