# Supplementary material for: Tunable Optical Metamaterial Enables Steganography, Rewriting, and Multilevel Information Storage
Source: Nanomicro Lett. 2025 Sep 5;18:58. doi: 10.1007/s40820-025-01897-9 (PMC12413373; doi:10.1007/s40820-025-01897-9)
Supplement: Supplementary file 1 — Supplementary file1 (DOCX 6224 kb) [file 40820_2025_1897_MOESM1_ESM.docx]

Supporting Information for

**Tunable Optical Metamaterial Enables Steganography, Rewriting, and Multilevel Information Storage**

Jianchen Zheng^1,2,&^, Yuzhao Zhang^3,4,&^, Haibo Yu^1,*^, Jingang Wang^1,2^, Hongji Guo^1^, Ye Qiu^1,2^, Xiaoduo Wang^1^, Yu Feng^5^, Lianqing Liu^1^ and Wen Jung Li^1,5,*^

^1^ State Key Laboratory of Robotics, Shenyang Institute of Automation, Chinese Academy of Sciences, Shenyang 110016, P. R. China

^2^ University of Chinese Academy of Sciences, Beijing 100049, P. R. China

^3^ School of Future Technology, Shanghai University, Shanghai 200444, P. R. China

^4^ Research Center of Micro/nano-Manipulation, Shanghai University, Shanghai 200444, P. R. China

^5^ Department of Mechanical Engineering, City University of Hong Kong, Hong Kong 999077, P. R. China

^&^ Jianchen Zheng and Yuzhao Zhangcontributed equally to this work and should be considered co-first authors.

* Corresponding authors. E-mail: [wenjli@cityu.edu.hk](mailto:wenjli@cityu.edu.hk) (Wen Jung Li); [yuhaibo@sia.cn](mailto:yuhaibo@sia.cn) (Haibo Yu)

**Table S1
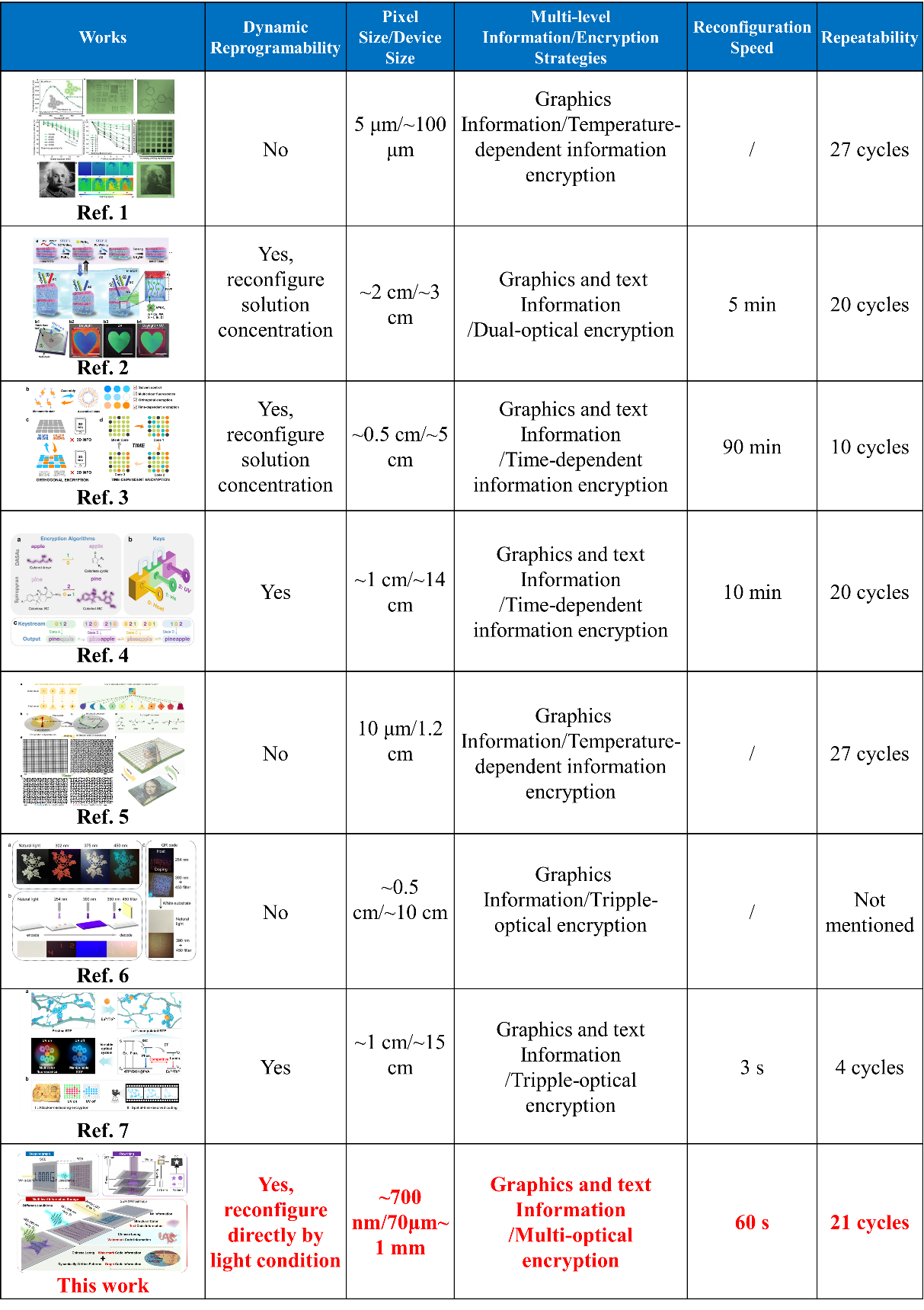
** Comparison of recently published optical encryption devices [S1-S7]

**Supplementary Figures**

**
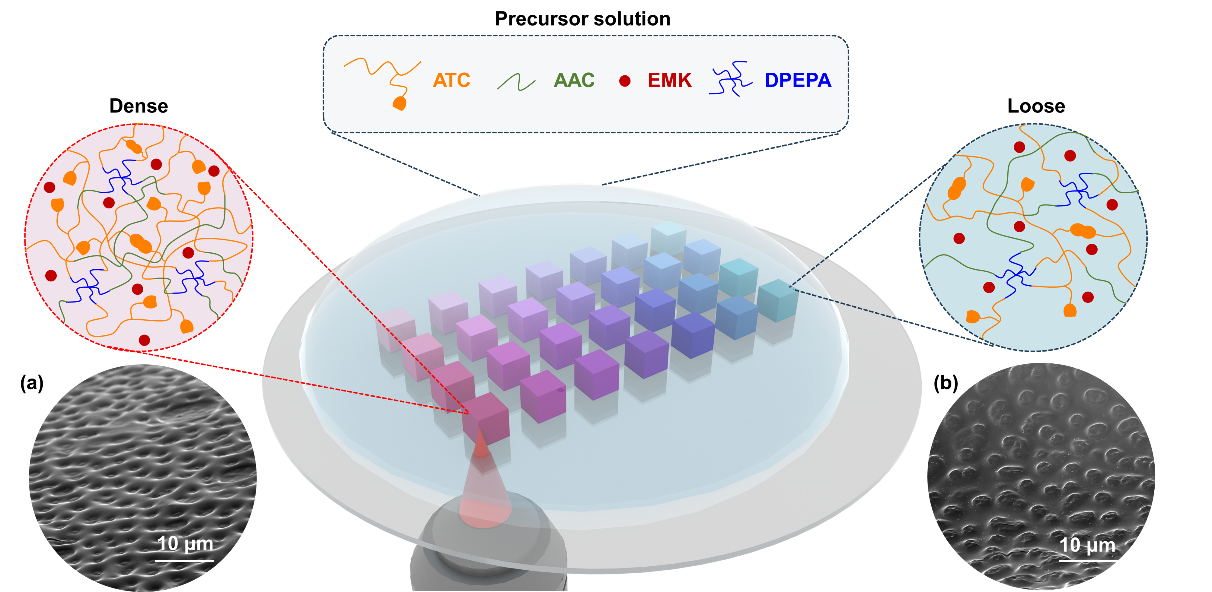
**

**Fig. 1** Different processing parameters affect the crosslink density of microstructures. The dense layer has a denser crosslinked network than the loose layer. (**a**) and (**b**) are their corresponding SEM characterization results, respectively. Scale bar: 10 μm

**Fig.**
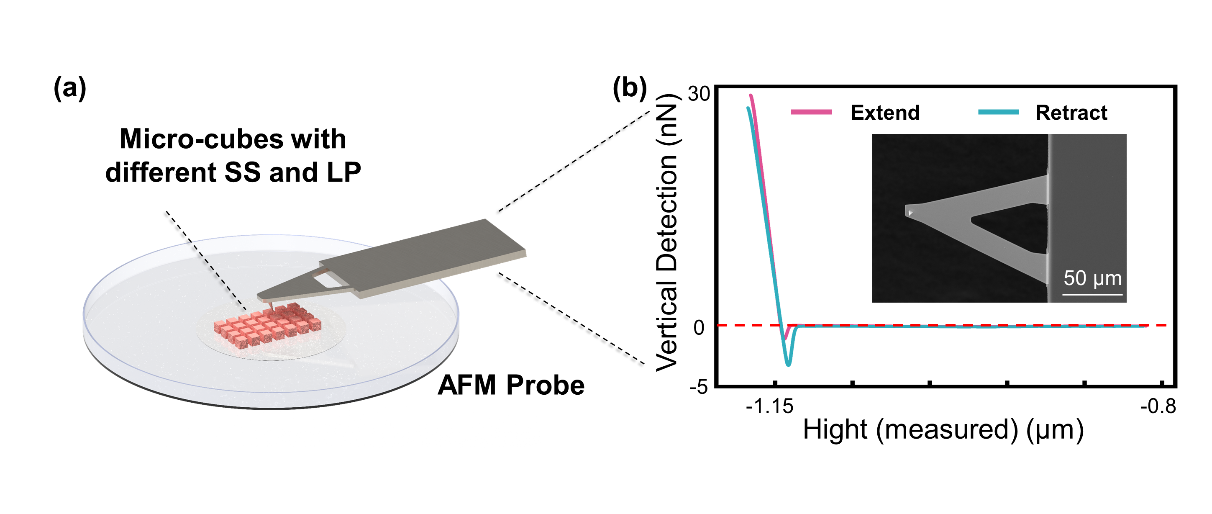
**2** Young's modulus measured by AFM. (**a**) Schematic diagram of the test. (**b**) Corresponding force diagram. The inset shows the SEM results of the probe. Scale bar: 50 μm

**Fig.**
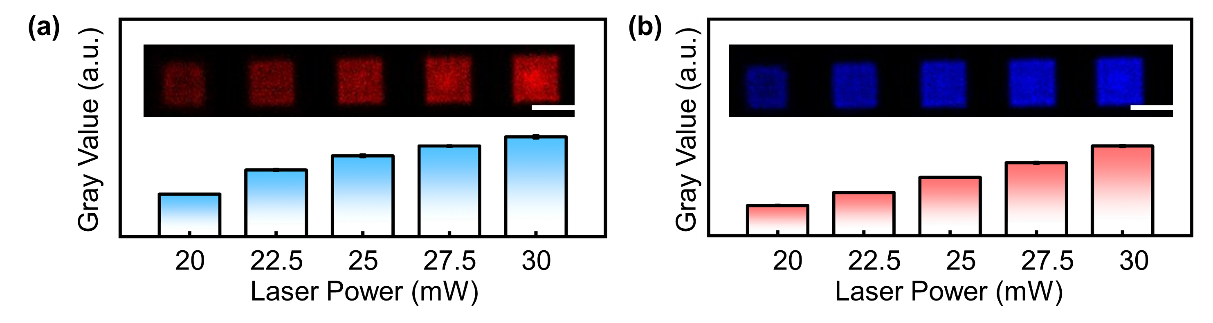
**3** Influence of laser power of fluorescence intensity. SS = 10 mm/s. (**a**) $\lambda_{ex}$ = 640 nm, $\lambda_{em}$ = 663 – 738 nm. (**b**) $\lambda_{ex}$ = 405 nm, $\lambda_{em}$ = 425 – 475 nm. Scale bar: 10 μm

**
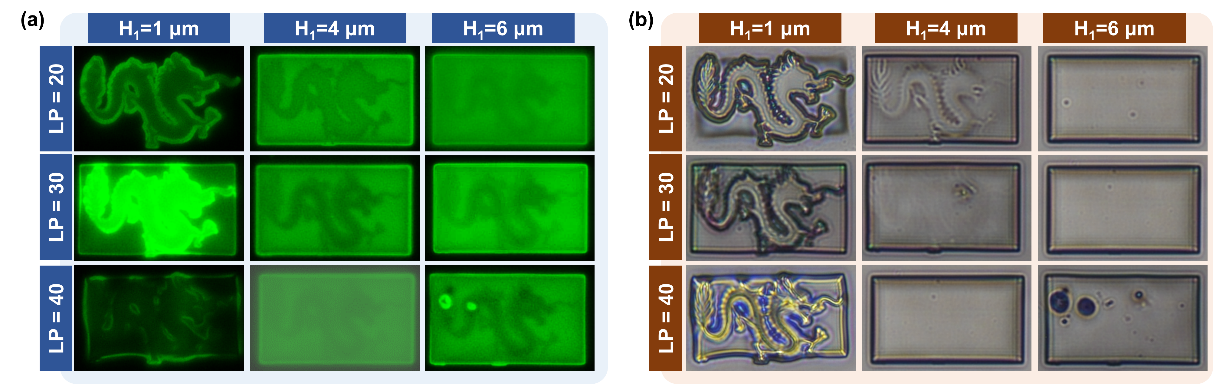
Fig. 4** Effects of different processing parameters on the display and encryption

**
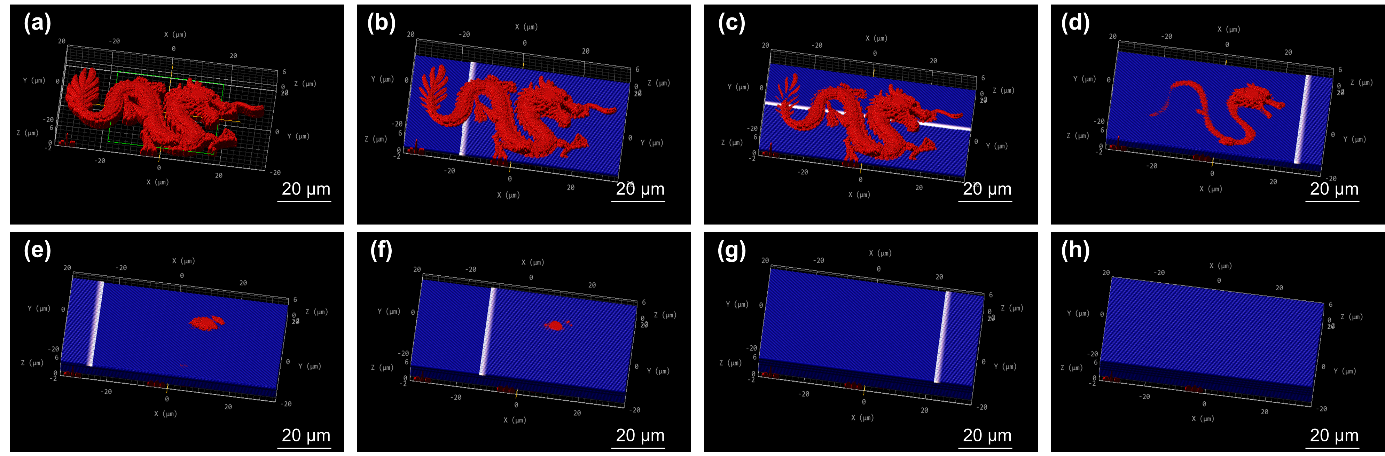
****Fig. 5** DLW's layer-by-layer scanning process of the “Chinese Loong” and encrypted layers

**
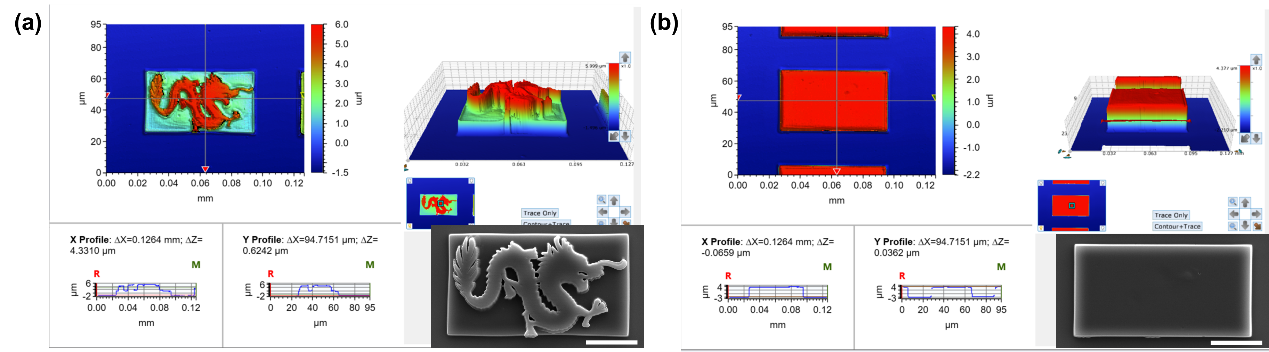
****Fig. 6** Three-dimensional surface morphology and SEM characterization of non-encrypted and encrypted modes. (**a**) Non-encrypted modes. (**b**) Encrypted modes. Scale bar: 20 μm


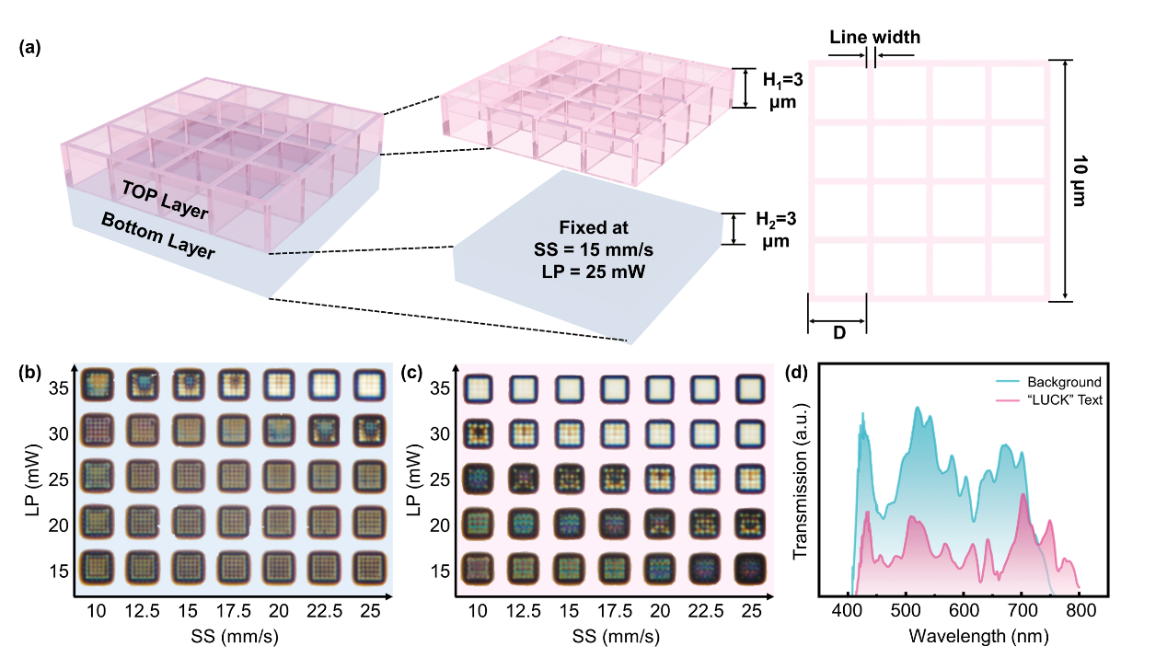


**Fig. 7** Effect of different parameters on structural color blocks(SCBs). (**a**) Design model for SCBs. (**b**) Optical pictures of SCB blocks processed with different processing parameters at D = 1.6. (**c**) Optical pictures of SCB blocks with different processing parameters at D = 2.3


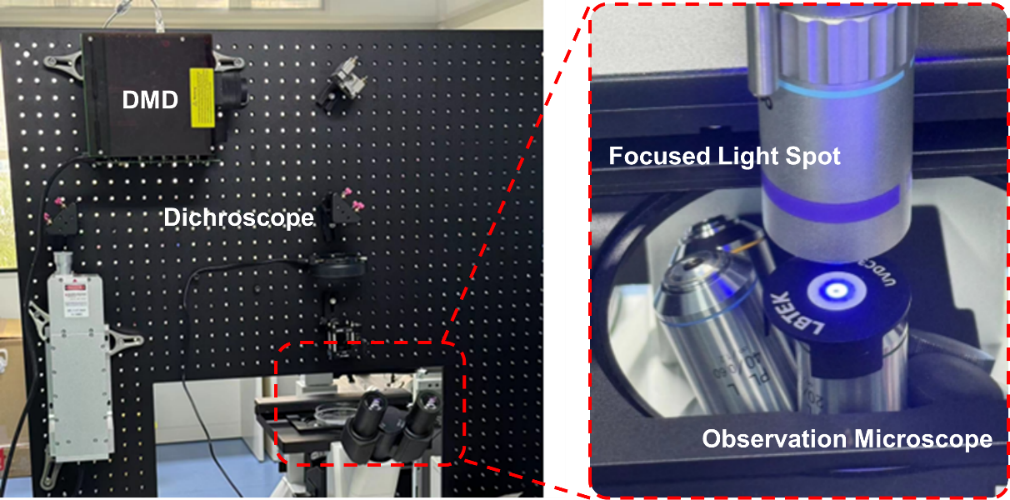


**Fig**. **8** Physical picture of a multi-light-field coupled in-situ control system

**
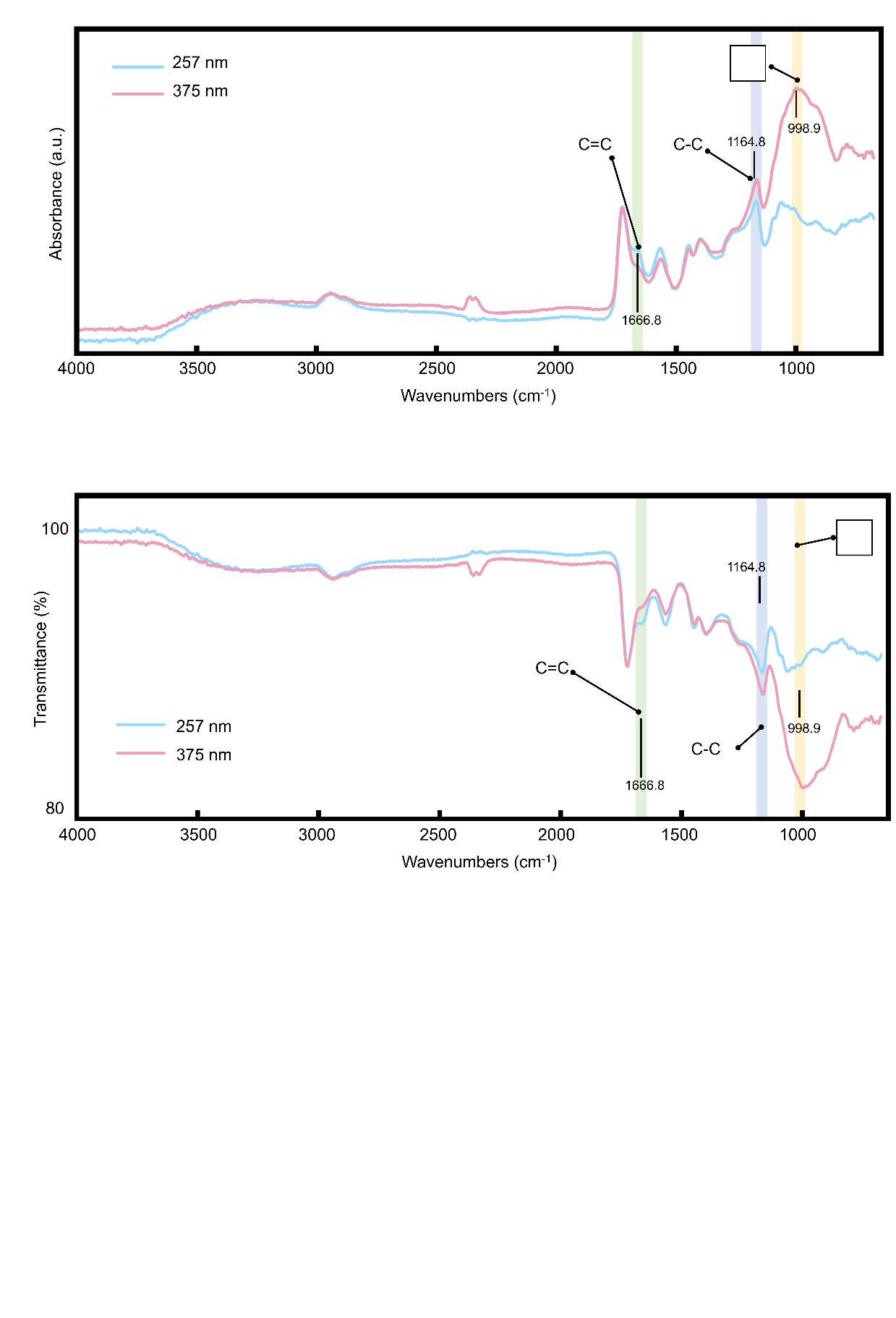
**

**Fig**. **9** Transmittance characterization of different laser irradiated structures by Micro-FTIR

**
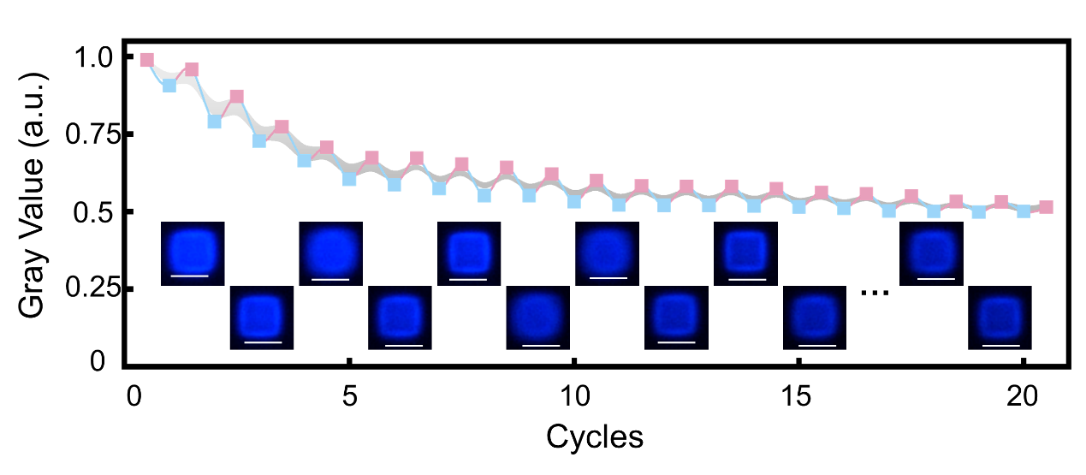
**

**Fig**. **10** Results of fluorescence gray scale characterization of structures undergoing cyclic laser irradiation

**
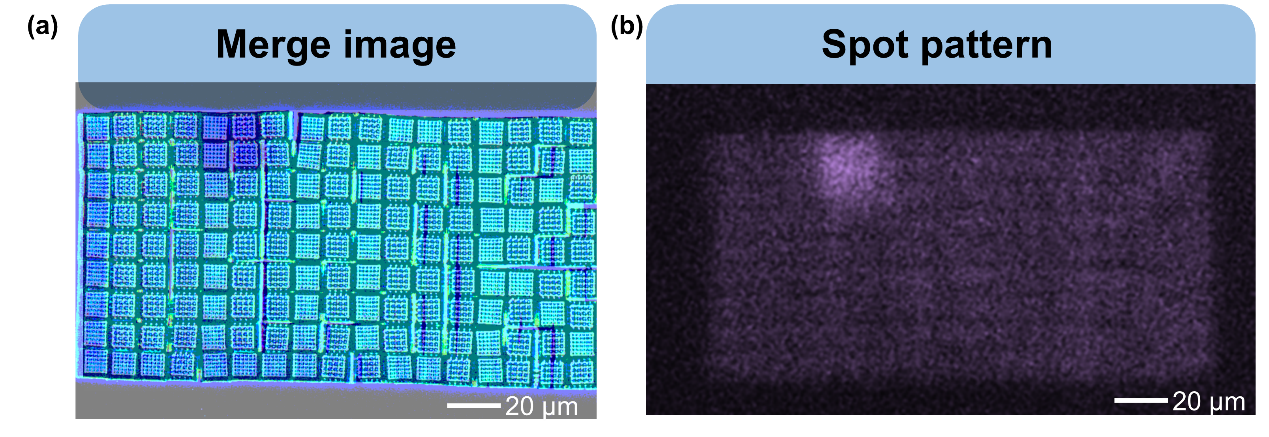
Fig**. **11** Merging the information in CH1 with the white light text information of μ-DMED to obtain “LOCK”. (**a**) Merge image. (**b**) Laser irradiated spot

**
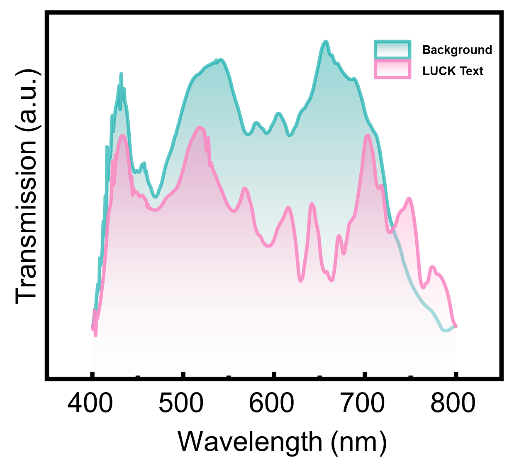
**

**Fig. 12** Spectral curves of SCB text messages after long-term storage

**Fig**.
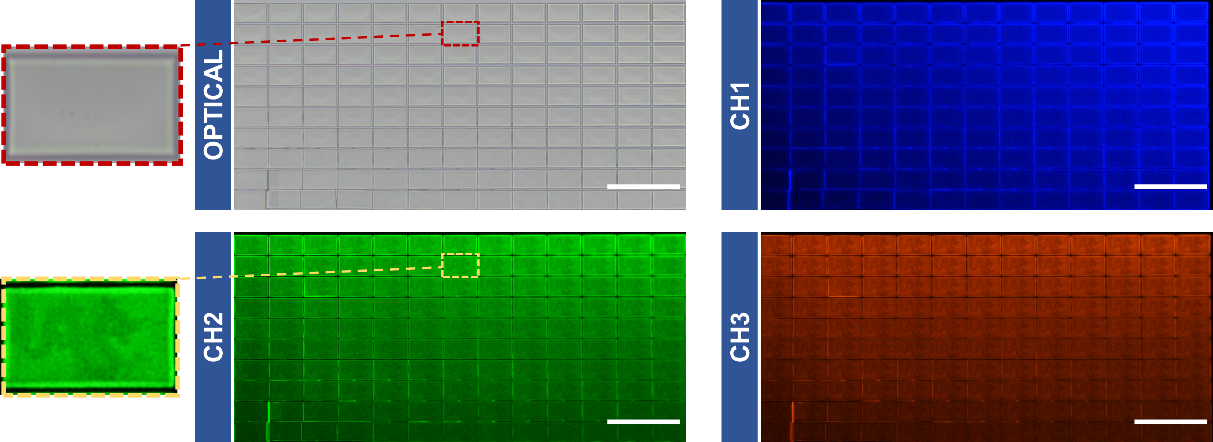
**13** Long-term preservation ability of the Chinese Loong watermark. Scale bar: 150 μm

**Supplementary Movies**

**Movies S1** Structural color blocks based processing

**Movies S2** Preparation process of large-scale encrypted displays based on fluorescent gray gradient blocks

**Supplementary References**

1. M. Zhang, Y. Lee, Z. Zheng, M.T.A. Khan, X. Lyu et al., Micro- and nanofabrication of dynamic hydrogels with multichannel information. Nat. Commun. **14**(1), 8208 (2023). <https://doi.org/10.1038/s41467-023-43921-9>
2. H. Han, J.W. Oh, H. Lee, S. Lee, S. Mun et al., Rewritable photoluminescence and structural color display for dual-responsive optical encryption (adv. mater. 14/2024). Adv. Mater. **36**(14), 2470100 (2024). <https://doi.org/10.1002/adma.202470100>
3. Q. Wang, B. Lin, M. Chen, C. Zhao, H. Tian et al., A dynamic assembly-induced emissive system for advanced information encryption with time-dependent security. Nat. Commun. **13**(1), 4185 (2022). <https://doi.org/10.1038/s41467-022-31978-x>
4. A. Gao, F. Sun, Y. Duan, Y. Zhang, X. Liu et al., Programmable encryption based on photochromism of spiropyrans and donor–acceptor stenhouse adducts. Adv. Funct. Mater. **34**(26), 2316457 (2024). <https://doi.org/10.1002/adfm.202316457>
5. M. Zhang, A. Pal, Z. Zheng, G. Gardi, E. Yildiz et al., Hydrogel muscles powering reconfigurable micro-metastructures with wide-spectrum programmability. Nat. Mater. **22**(10), 1243–1252 (2023). <https://doi.org/10.1038/s41563-023-01649-3>
6. J. Jin, Y. Wang, K. Han, Z. Xia, Rigid phase formation and Sb^3+^ doping of tin (IV) halide hybrids toward photoluminescence enhancement and tuning for anti-counterfeiting and information encryption. Angew. Chem. Int. Ed. **63**(33), e202408653 (2024). <https://doi.org/10.1002/anie.202408653>
7. L. Li, J. Zhou, J. Han, D. Liu, M. Qi et al., Finely manipulating room temperature phosphorescence by dynamic lanthanide coordination toward multi-level information security. Nat. Commun. **15**(1), 3846 (2024). <https://doi.org/10.1038/s41467-024-47674-x>
